# Real-Time Whole-Body Visualization of Chikungunya Virus Infection and Host Interferon Response in Zebrafish

**DOI:** 10.1371/journal.ppat.1003619

**Published:** 2013-09-05

**Authors:** Nuno Palha, Florence Guivel-Benhassine, Valérie Briolat, Georges Lutfalla, Marion Sourisseau, Felix Ellett, Chieh-Huei Wang, Graham J. Lieschke, Philippe Herbomel, Olivier Schwartz, Jean-Pierre Levraud

**Affiliations:** 1 Institut Pasteur, Macrophages et Développement de l'Immunité, Department of Developmental and Stem Cells Biology, Paris, France; 2 CNRS URA2578, Paris, France; 3 Université Pierre et Marie Curie, Paris, France; 4 Institut Pasteur, Virus et Immunité, Department of Virology, Paris, France; 5 CNRS URA3015, Paris, France; 6 CNRS UMR5235, Dynamiques des Interactions Membranaires et Pathologiques, Montpellier, France; 7 Université Montpellier II, Montpellier, France; 8 Australian Regenerative Medicine Institute, Monash University, Clayton, Victoria, Australia; University of North Carolina at Chapel Hill, United States of America

## Abstract

Chikungunya Virus (CHIKV), a re-emerging arbovirus that may cause severe disease, constitutes an important public health problem. Herein we describe a novel CHIKV infection model in zebrafish, where viral spread was live-imaged in the whole body up to cellular resolution. Infected cells emerged in various organs in one principal wave with a median appearance time of ∼14 hours post infection. Timing of infected cell death was organ dependent, leading to a shift of CHIKV localization towards the brain. As in mammals, CHIKV infection triggered a strong type-I interferon (IFN) response, critical for survival. IFN was mainly expressed by neutrophils and hepatocytes. Cell type specific ablation experiments further demonstrated that neutrophils play a crucial, unexpected role in CHIKV containment. Altogether, our results show that the zebrafish represents a novel valuable model to dynamically visualize replication, pathogenesis and host responses to a human virus.

## Author Summary

Chikungunya, a re-emerging disease caused by a mosquito-transmitted virus, is an important public health problem. We developed a zebrafish model for chikungunya virus infection. For the first time, rise and death of virus-infected cells could be live imaged in the entire body of a vertebrate. We observed a widespread wave of apparition of newly infected cells during the first day after inoculation of the virus. We then found that infected cells died at a strongly organ-dependent rate, accounting for the progressive shift of virus localization. Notably, the virus persisted in the brain despite apparent recovery of infected zebrafish. We found this recovery to be critically dependent on the host type I interferon response. Surprisingly, we identified neutrophils as a major cell population expressing interferon and controlling chikungunya virus.

## Introduction

Chikungunya virus (CHIKV) is a mosquito-transmitted virus that causes serious illness and has reemerged in Africa and Asia since 2000, causing outbreaks with millions of cases after decades of near-absence [1]. The epidemic spread to previously CHIKV-free areas, such as La Reunion Island in the Indian Ocean, probably as a consequence of the adaptive mutation of the virus to a new vector species, *Aedes albopictus*, the tiger mosquito [2], [3], [4], [5]. Unlike traditional CHIKV vectors such as *A. aegypti*, *A. albopictus* can produce cold-resistant eggs and is a major invasive species of temperate countries [6], and as it also seems to better transmit the virus [7], CHIKV is now threatening to invade many new territories including the Caribbean, southeast USA and southern Europe. There is currently no commercial vaccine or efficient treatment available for this disease [1].

CHIKV infection is often debilitating and may last from weeks to months; its symptoms in humans include acute fever, rash, joint and muscle pain, chronic arthralgia and, more rarely, severe complications with a fatality rate of about 1 in 1000 [1], [8], [9], [10]. However, CHIKV infection in humans is generally self-limiting, with a short but intense viremia lasting about one week, controlled by type-I interferons (IFNs) [8]. Specific antibodies become detectable shortly after and contribute to virus clearance [11].

CHIKV tropism *in vivo*, and host innate immune responses are only starting to be characterized [8], [9]. In humans, the virus displays a wide cellular tropism *in vitro*, infecting fibroblasts, endothelial, epithelial, muscle cells, and to a lower extent, myeloid cells like macrophages [12], [13]. Severe encephalopathies have been reported in CHIKV-infected humans, mostly in infants - more than half infected newborns [14], compared with ∼0.1% in adults [15] - yet CHIKV neurotropism remains controversial [16], [17]. It is still debated whether CHIKV may persist in some cellular reservoirs after the early viremic phase and be responsible for painful relapses that may persist for months.

Murine and macaque models that recapitulate to some extent the human disease have been developed [18], [19], [20], [21]. These models have greatly improved our understanding of the disease, but they do not allow the visualization of infection dynamics and host antiviral and inflammatory responses at the whole body level.

Recently, the zebrafish *Danio rerio* has emerged as a new model for host-pathogen interactions, largely because their small, transparent larvae are highly suited to *in vivo* imaging. Zebrafish possess an innate and adaptive immune system akin to that of mammals, but its free-swimming larva relies solely on innate immunity for the first month of its life, allowing the specific dissection of innate immune responses [22]. At the larval stage, cellular immunity consists of myeloid cells only, with neutrophils and macrophages being the main effector cells [23], [24]. As in mammals, antiviral immunity is orchestrated by virus-induced IFNs, of which the zebrafish possess four (IFNφ1-4) [25], [26], structurally similar to mammalian type I IFNs [27]. Zebrafish type I IFNs have been divided into two groups: I (IFNφ1 and φ4) and II (IFNφ2 and φ3), that signal via two different heterodimeric receptors, CRFB1/CRFB5 and CRFB2/CRFB5, respectively. As IFNφ2 is expressed only in adults and IFNφ4 has little activity, the IFN response is mediated by IFNφ1 and IFNφ3 in zebrafish larvae [26], [28].

Since CHIKV infects both mammals and insects, and since other members of the alphavirus genus naturally infect salmonids [29], [30], we hypothesized that the zebrafish free-swimming larva might be sensitive to CHIKV, allowing live imaging of infected cells and dynamics of host-virus relationship in the entire animal. Here we describe a new CHIKV infection model in zebrafish larvae and analyze the dynamics of infection, cell death and host responses. Type I IFNs were critical for survival of CHIKV-infected zebrafish and we identified an unexpected role for neutrophils in both the production of type I IFNs and control of CHIKV infection.

## Results

### CHIKV infects zebrafish larvae

**Figure 1:**
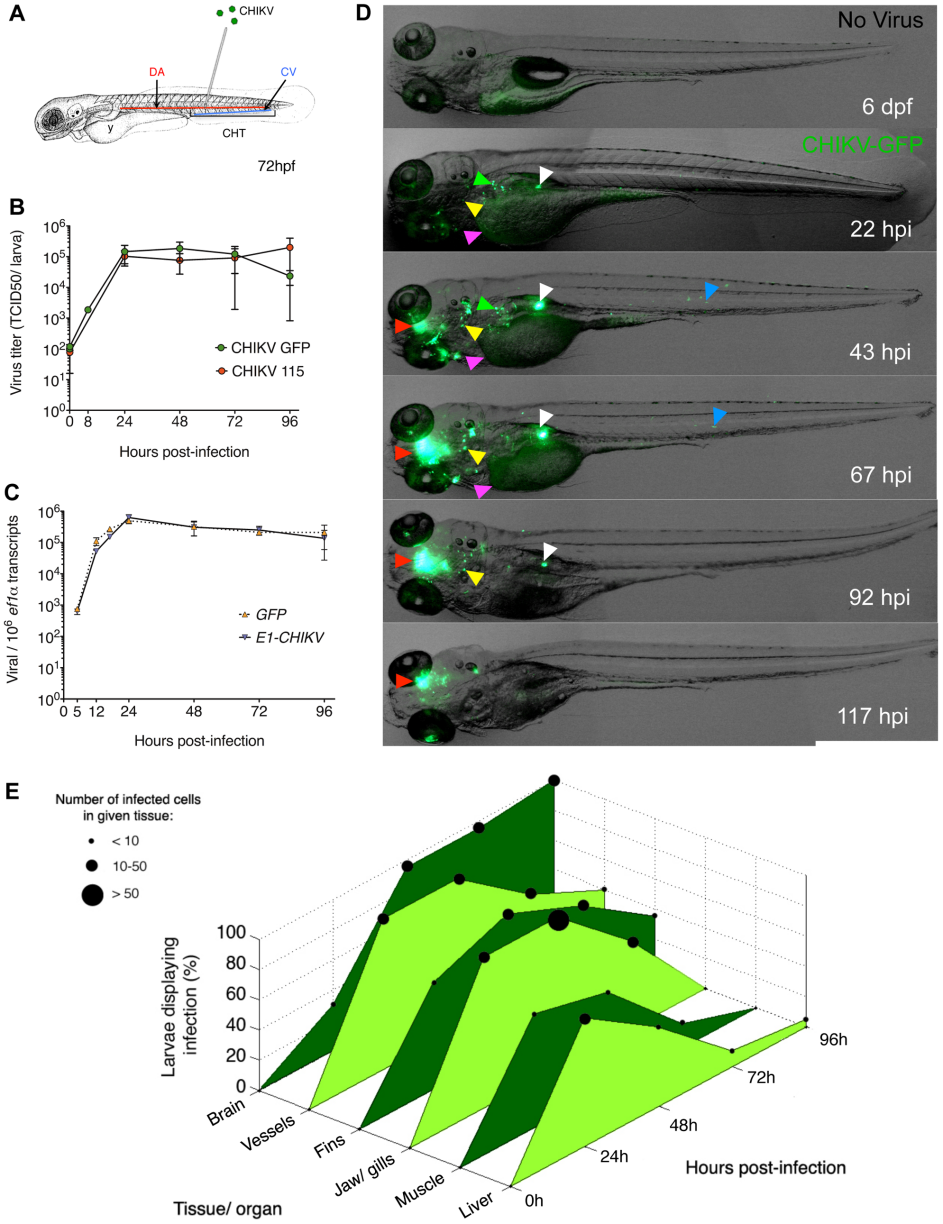
CHIKV replicates in zebrafish and disseminates to various organs. (A) Scheme of a 72 hours post-fertilization (hpf) larva, showing the site of injection in the dorsal aorta (DA) or caudal vein (CV), the caudal hematopoietic tissues (CHT) and the yolk syncitial cell (y). (B) Virus titers in zebrafish larvae infected with wild-type CHIKV-115 or with CHIKV-GFP. Data represent mean ± s.e.m of 2–5 pools of 4 larvae from 5 independent experiments. (C) qRT-PCR of viral *E1* and *GFP* transcripts after CHIKV-GFP infection. Mean ± s.e.m of 3 pools of 10 larvae from 3 independent experiments. (D) Overlay of transmission and green fluorescence stereomicroscope images of a single representative wild-type CHIKV-GFP-infected larva, live imaged at different hours post infection (hpi). CHIKV infection is shown in the brain, liver, head mesenchyme, muscle, swim bladder and yolk (red, green, yellow, blue, white and magenta arrowheads, respectively). (E) Assessment using fluorescence stereomicroscopy of penetrance (% of infected larvae displaying infection) and severity (number of cells) of infection in specific organs at different time-points after CHIKV-GFP infection, following immunohistochemistry (IHC) with an anti-capsid antibody. Data pooled from 2 independent experiments, *N* = 20 larvae for each time-point.

We first asked whether zebrafish were sensitive to CHIKV infection. Larvae aged 3 days post-fertilization (dpf) were injected intravenously (Figure 1A) with ∼10^2^ TCID50 CHIKV, using a strain from the 2005–2006 Reunion Island outbreak (CHIKV-115) [13] or a closely related strain engineered to express GFP (CHIKV-GFP) [3]. Both CHIKV-115 and CHIKV-GFP established infection and replicated *in vivo*, with production of infectious virions peaking at 24–48 hours post-infection (hpi) (>10^5^ TCID50/larva; i.e., >10^8^ TCID50/gram of tissue) (Figure 1B). Using qRT-PCR with E1-specific primers, we found similar kinetics (Figure 1C). These primers amplify both the genomic and subgenomic transcripts, hence mainly reflect the level of the latter, which is more abundant in alphavirus-infected cells [31], although the ratio of genomic to subgenomic transcripts may vary widely among alphaviruses. Predictably, similar kinetics were obtained for virus-encoded, subgenomic promoter-driven *GFP* transcripts (Figure 1C). Symptoms, most obvious at 3 days post-infection (dpi), were mild compared to other zebrafish viral infection models [28], [32], [33], [34], the most consistent one being opacification of the yolk (Figure S1A in Text S1). Other less frequent signs included delay in swim bladder inflation, slowing of blood flow, irregular heartbeat, edema, loss of equilibrium and sluggish response to touch (Table S1 in Text S1). These signs were generally transient and by 5 dpi, >90% of infected larvae had apparently recovered, surviving until at least 7 dpi *(not shown)*.

### CHIKV infection is cleared in most tissues but infection persists in brain parenchyma

We monitored organs and cells of live CHIKV-GFP infected zebrafish. GFP patterns varied through time (Figure 1D) and between individuals (Figure S1B in Text S1). GFP was detected in liver, jaw, gills, vascular endothelium, eyes, fins, blood cells, muscle fibers, brain, spinal cord, swim bladder and the yolk syncytial layer. Similar patterns were observed in CHIKV-115 infected zebrafish after fixation and immunohistochemistry (IHC) with a capsid-specific antibody *(not shown)*. We quantified the distribution of infected cells in the entire organism over time to establish the kinetics of viral dissemination (Figure 1E). The amount of infected cells peaked by 1–2 dpi in most organs (jaw, fins, liver, vessels, musculature). This peak was followed by a sharp decrease both in the frequency of larvae showing infection in a given organ, and the number of infected cells per organ. By 4 dpi, CHIKV was cleared from most organs. In contrast, infection in the brain parenchyma became visible at 2 dpi in most animals and persisted at least until 5 dpi (Figures 1D and 1E), suggesting that the brain may represent a viral reservoir in zebrafish. At 7 dpi, the latest time point testable, infection in the brain was still strong (Figure S1C in Text S1); in addition, double staining of CHIKV-GFP infected larvae with anti-GFP and anti-capsid antibodies showed that almost all capsid-positive cells also expressed GFP, indicating that GFP expression was a reliable indicator of the infection, even into late stages.

**Figure 2:**
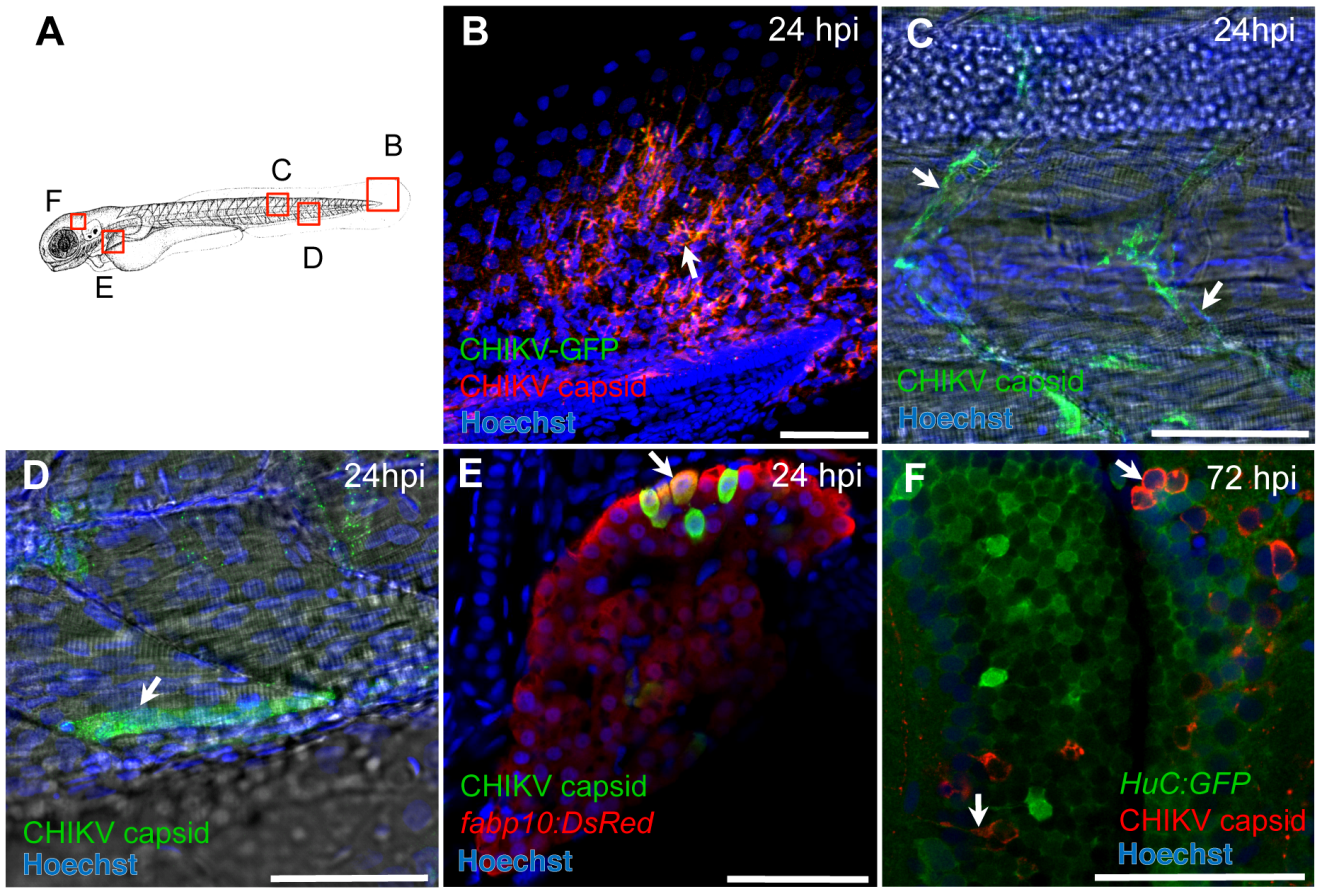
Cellular tropism of CHIKV. (A) Scheme of imaged regions in (B–F). (B–F) Confocal images of IHC-processed zebrafish at 24 hpi with CHIKV-GFP (B–E) or at 72 hpi with CHIKV-115 (F). As for all images, anterior to left, dorsal to top; scale bars, 50 µm. GFP staining in green in (B, F) DsRed staining in red in (E), capsid staining in red (B, F) or green (C–E); nuclei counterstained in blue. The *fabp10:dsRed* transgene labels hepatocytes, and *HuC:GFP*, post-mitotic neurons. Arrows show infection in fin fibroblasts (B), endothelial cells (C), a muscle fiber (D), hepatocytes (E) and neurons (F).

Confocal imaging of IHC-labeled CHIKV-infected larvae showed infection in various cell types (Figure 2A), namely fibroblasts in fins (Figure 2B) and jaw (*not shown*), endothelial cells (Figure 2C), muscle fibers (Figure 2D) and hepatocytes (Figure 2E, and Figure S2 in Text S1). Infection also occurred occasionally in red blood cells (Figure S2 in Text S1) but not in macrophages or neutrophils (*not shown*). In zebrafish brain, CHIKV was detected in both neurons and glial cells (Figure 2F, and Figure S2 in Text S1).

### Differential infected cell survival accounts for viral persistence in brain

**Figure 3:**
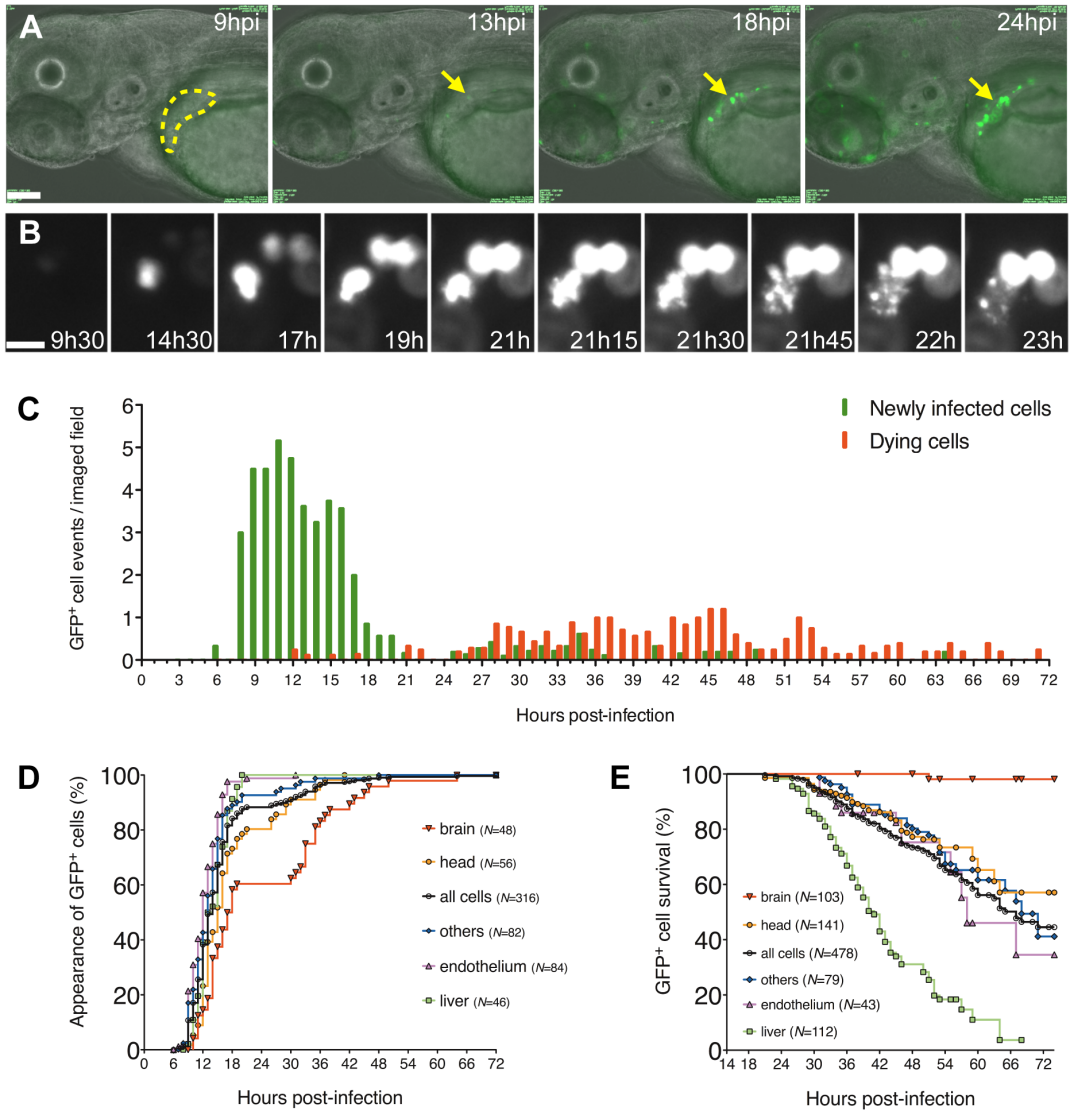
Kinetics of appearance and death of infected cells, from *in vivo* time-lapse imaging of CHIKV-GFP infection. (A, B) Movie frames showing emergence of infected cells (GFP^+^) in the liver and death of one cell during the first day of infection. Time post-infection (in hours and minutes) overlaid on images. (A) Entire field, overlay of transmission and GFP fluorescence (green), scale bar 100 µm. Liver delineated in yellow; arrows point to an hepatocyte becoming infected and dying. (B) Detail from the same movie, GFP fluorescence only, scale bar 20 µm, showing the rise and death of this infected hepatocyte. (C) Timings of appearance (green bars) and death (red bars) of immobile CHIKV-GFP infected cells, all organs pooled; (D, E) sub-analysis of the same dataset, showing kinetics of appearance (D) and death (E) of GFP^+^ cells per organ, displayed as Kaplan-Meier plots. *N* = Number of cells followed in each organ. In (C–E) data pooled from five independent experiments, with a total 24 fish imaged for 6–24 hours each, 4–8 animals per time-point.

To assess the dynamics of CHIKV infection and its cytopathic effects, we performed time-lapse imaging of CHIKV-GFP infected larvae (Figures 3A and 3B) and compiled the appearance and death of GFP^+^ cells (Figures 3C–E). 88% of newly infected cells appeared before 24 hpi in one major wave (Figure 3C). The median time of appearance of new GFP^+^ cells was 14±2 hpi with similar kinetics in all cell types (Figure 3D). Death of GFP^+^ infected cells presented apoptosis features such as membrane blebbing and cellular fragmentation (Figure 3B and Movies S1 and S2). It was frequent from 24 hpi onwards (Figure 3C), with an overall median death time of 67±4 hpi, but dependent on cell type (Figure 3E). For instance, liver cells were highly susceptible to CHIKV cytopathic effects, with a median occurrence of death at 41±5 hpi, implying that hepatocytes survive for ∼27 h following GFP detection, compared to a ∼53 h survival period for the general cell population. In contrast, almost all infected brain parenchyma cells survived at least until 72 hpi. These results demonstrate that the apparent shifting tropism of infection towards brain (Figure 1E) is largely due to differential cell survival.

### A protective type I IFN response is induced upon CHIKV infection

**Figure 4:**
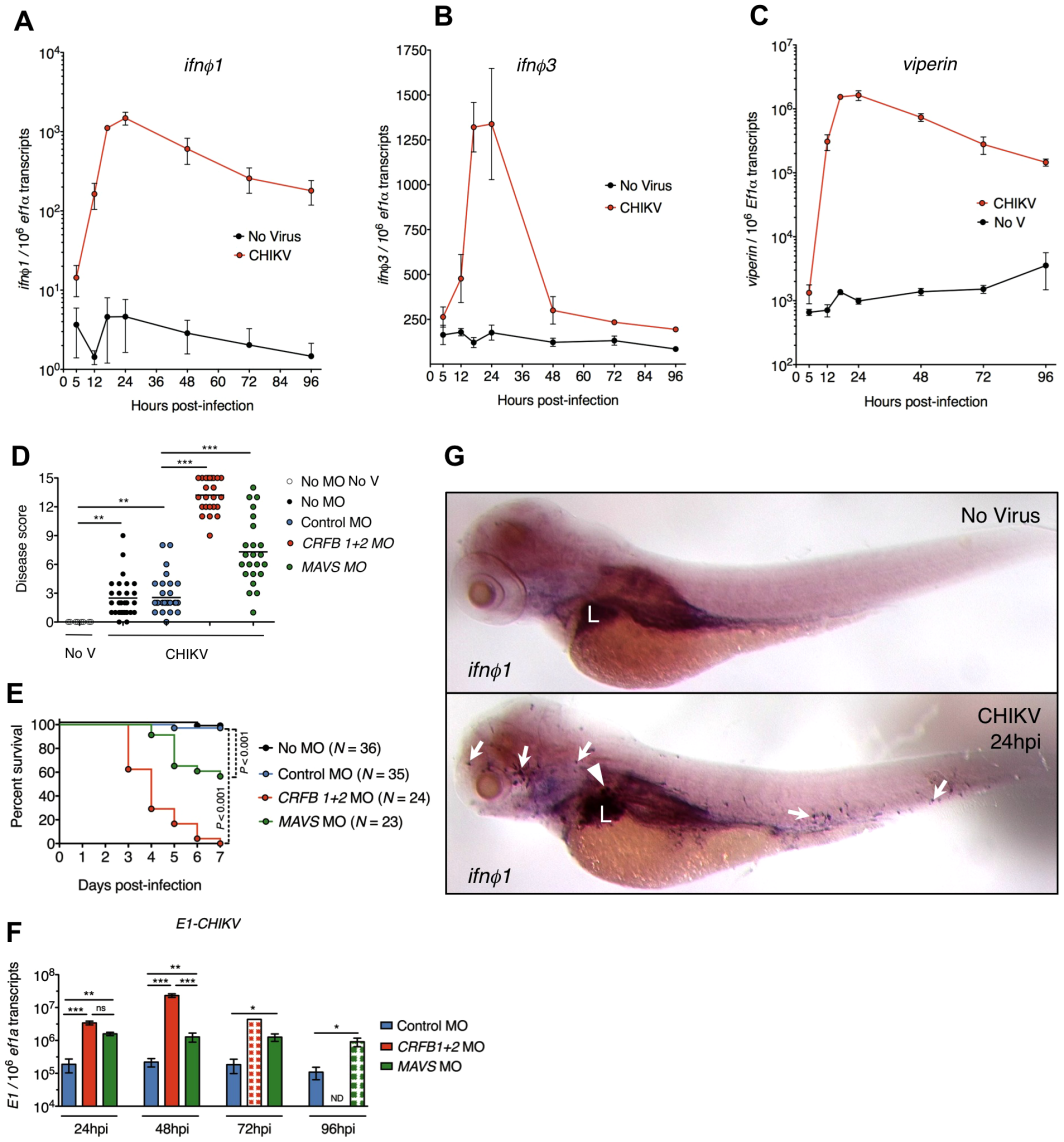
A protective interferon response is induced upon CHIKV infection. (A–C) Expression of zebrafish type I IFNs *ifnφ1* (A) and *ifnφ3* (B) and the IFN-stimulated gene *viperin* (C), upon CHIKV-GFP infection. qRT-PCR, mean ± s.e.m of 3 pools of 10 larvae from 3 independent experiments. (D–F) Effect of morpholino-mediated knockdown of IFN receptor subunits (*CRFB1+2* MO) and of MAVS (*MAVS* MO) on CHIKV-GFP infection. No MO, not injected with a morpholino; No V, uninfected controls; Control MO, injected with a unspecific morpholino oligonucleotide. (D) Disease score at 3 days post-infection; (E) survival of infected zebrafish; (F) quantification of viral *E1* transcripts over time. qRT-PCR, mean ± s.e.m of 3 pools of 3–5 larvae, except for the CRFB morphants at 72 hpi (one single pool of 5 larvae). Hatched bars represent groups where a fraction of the fish had already died, implying selection of survivors for the analysis. ND, not determined. (G) Pattern of *ifnφ1* expression, whole-mount *in situ* hybridization in uninfected larva (top) or CHIKV-GFP infected larva (bottom) at 24 hpi, representative out of 7 fish. Arrows indicate some *ifnφ1^+^* leukocytes, arrowhead point to an *ifnφ1^+^* hepatocyte; L = liver. (***P < 0.001; **P < 0.01; *P < 0.05; ns - not significant).

Type I IFN signaling is critical for control of CHIKV in mammals [18], [20]. In zebrafish larvae, CHIKV triggered high mRNA levels of *ifnφ1* (NM_207640, secreted isoform transcript) and *ifnφ3* (NM_001111083), and of various IFN-induced genes including *viperin/vig-1/rsad2* (NM_001025556) (Figures 4A–C and *not shown*). *Ifnφ1* and *viperin* levels, peaking at 17–24 hpi, remained high for at least 4 days, correlating with viral burden. These levels were higher than previously observed with fish viruses in zebrafish [28], [32], [34]. *Ifnφ3* induction was less prominent in breadth and duration. To assess the role of the IFN response, we knocked down receptors for all IFNφs with antisense morpholino oligonucleotides (MO) directed to the CRFB1 (NM_001079681) and CRFB2 (NM_001077626) subunits [26]. When IFN receptor expression was impaired (CRFB1+2 MO), the disease was particularly severe (Table S1 in Text S1), as measured by a disease score (*defined in* Table S2 in Text S1) (Figure 4D). Among CRFB1+2 morphant fish, >90% died from infection (Figure 4E), while virus burden was increased up to 100-fold when compared to infected control morphants (Figure 4F). Upstream of IFN signaling, sensing of CHIKV through the cytosolic pathway was important as knockdown of MAVS (IPS-1/CARDIF/VISA) (NM_001080584) (Figure S3 in Text S1) also led to an increase in disease severity and mortality, as well as in virus burden (Figures 4D–F), consistent with results obtained in mice [35], [36]. As expected, knockdown of CRFB1 and CFRB2 did not affect *ifnφ1* production but blocked *viperin* expression, whereas in MAVS morphants, both *ifnφ1* and *viperin* levels were significantly reduced (Figure S3 in Text S1). Altogether, these results show that the type I IFN pathway controls CHIKV replication and pathogenesis in zebrafish.

### Zebrafish *ifnφ1:mCherry* transgene labels IFN-producing cells

**Figure 5:**
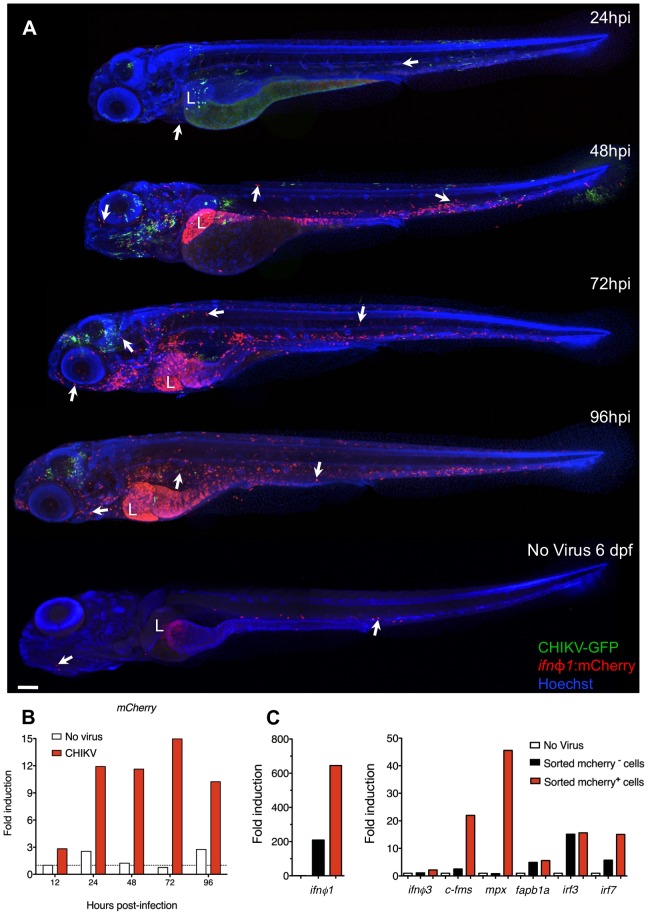
*Ifnφ1*-expressing cells are leukocytes and hepatocytes. (A) Distribution of *ifnφ1*-expressing cells revealed by the *ifnφ1:mCherry* reporter transgene. IHC, mCherry stained in red, GFP in green, nuclei in blue. Confocal imaging, reconstructed composite images of maximal projections to cover the whole body. Representative examples of CHIKV-GFP-infected fish at different time points after infection. Below is an uninfected control at the equivalent of 72 hpi. Arrows indicate some mCherry^+^ leukocytes, L = liver. Scale bar, 100 µm. (B) qRT-PCR of *mCherry* (normalized to *ef1α*) upon CHIKV-GFP infection in *ifnφ1:mCherry* fish. Fold induction to uninfected fish at 12 hpi; data for one pool of 10 larvae per time point. (C) Expression profile of FACS-sorted cells from CHIKV-infected *ifnφ1:mCherry* fish (3 dpi). qRT-PCR, fold induction compared to entire uninfected fish (No Virus). Data representative of 2 independent experiments.

To identify the source of IFN, we first performed whole-mount in situ hybridization (WISH) using an antisense probe for *ifnφ1* at the peak of the response. In CHIKV-infected larvae, *ifnφ1* expression was detected in the liver and in scattered cells with a morphology and distribution evoking leukocytes (Figure 4G). To better visualize the spatiotemporal dynamics of IFN production, we designed a transgenic IFNφ1 reporter zebrafish, in which the *ifnφ1* promoter drives expression of the mCherry red fluorescent protein. In uninfected 3–6 dpf transgenic larvae, mCherry was detected in very few (10–30) cells, all with leukocyte morphology and mostly residing in the caudal hematopoietic tissue (CHT), but upon CHIKV infection, the number of mCherry^+^ cells dramatically increased (Figure 5A). Starting from 2 dpi, two main populations of mCherry^+^ cells were detected: hepatocytes and motile leukocytes (Figure 5A and Movie S3). The mCherry^+^ leukocytes were dispersed throughout the body except the CNS, mostly in the anterior region and the CHT, and persisted until at least 4 dpi. This pattern of expression of the reporter transgene was similar to that of the endogenous *ifnφ1* gene (Figure 4G), but appearing later, a delay apparently due to the time required for protein expression and maturation, since at 24 hpi mCherry fluorescence was still low despite *mCherry* mRNA expression (Figure 5B). Thus, the reporter transgene is faithful but somewhat delayed compared to endogenous *ifnφ1*. Notably though, viral GFP and mCherry were not detected in the same cells, suggesting that IFN release occurs mostly in uninfected or non-productively infected cells.

### Neutrophils are the main IFN-producing leukocyte population upon CHIKV infection

We further characterized IFN-producing cells. We FACS-sorted mCherry^+^ cells from infected *ifnφ1:mCherry* zebrafish at 3 dpi and analyzed their mRNA expression profile (Figure 5C). As expected, expression of *ifnφ1* was highest in sorted mCherry^+^ cells. These cells did not notably co-express *ifnφ3*. Among leukocyte genes, the macrophage marker *c-fms/csf1r* (NM_131672) was increased in mCherry^+^ cells, but the strongest enrichment was for myeloperoxydase (*mpx*, NM_212779), a specific neutrophil marker in zebrafish [23], [24]. The hepatocyte marker *fabp1a* (NM_001044712) was also expressed, consistent with some hepatocytes producing IFN. Sorted mCherry^−^ cells expressed lower but significant *ifnφ1* levels – especially if compared to naïve larvae, which express it to an extremely low level -, likely due to the aforementioned delay. Both mCherry^+^ and mCherry^−^ expressed the IFN-inducing transcription factors *irf3* (NM_001111083) and *irf7* (NM_200677), with the latter being enriched among mCherry^+^ cells.

**Figure 6:**
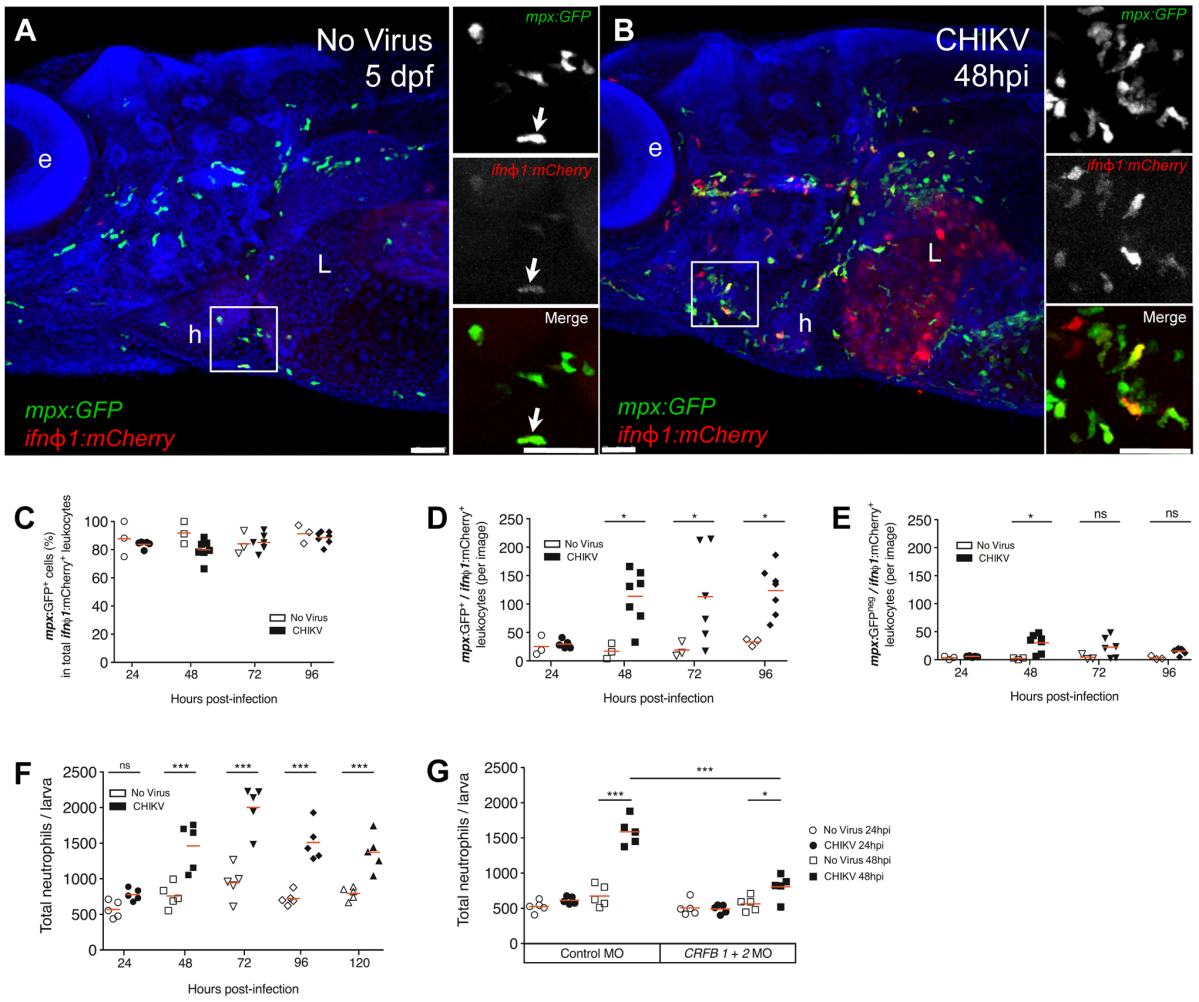
*Ifnφ1*-expressing leukocytes are mostly neutrophils, which increase in an IFN-dependent manner. (A–E) IHC of anterior region of *mpx:GFP/ifnφ1:mCherry* double transgenic fish. (A–B) Confocal imaging of an uninfected (A) and CHIKV-115-infected (B) larva at 48 hpi, maximal projection, scale bar 50 µm, mCherry staining in red, GFP staining in green, nuclei in blue (e: eye; L: liver; h: heart); on the right, single color and merged images of the detailed square. (C) Percentage of neutrophils (GFP^+^) among mCherry^+^ leukocytes, per field. (D) Number of mCherry^+^ neutrophils (GFP^+^) per field. (E) Number of other mCherry^+^ leukocytes (GFP^−^) per field. For (C–E), *N* = 3 (No Virus) or *N* = 5–7 (CHIKV). (F, G) Uninfected or CHIKV-GFP-infected larvae were stained with Sudan Black B to reveal myeloperoxidase granules. Total neutrophil numbers per individual zebrafish were quantified using a stereomicroscope. (F) Neutrophil numbers over time in standard (no morpholino treatment) animals; (G) Neutrophils numbers in interferon receptor knockdown fish (*CRFB1+2* MO) compared to control morphants. *N* = 5 fish per group (***P < 0.001; **P < 0.01; *P < 0.05; ns - not significant).

To confirm the involvement of neutrophils, we crossed neutrophil reporter *mpx:GFP* with *ifnφ1:mCherry* zebrafish. In double transgenic CHIKV-infected zebrafish, either uninfected or CHIKV-infected, more than 80% of mCherry^+^ leukocytes expressed GFP (Figures 6A–C). Their morphology, distribution, speed, and presence of refractile moving granules, as assessed by live Nomarski microscopy, were also consistent with neutrophil identity [24] *(not shown)*.

The number of mCherry^+^ neutrophils strongly increased by 48 hpi and remained high until at least 96 hpi (Figure 6D, and Figure S4A in Text S1). Other mCherry^+^ leukocytes (mostly *mpeg1* ^+^ macrophages, *not shown*) were also increased at 48 hpi, but in lower numbers, and notably in the CHT where they transiently made up about half the mCherry^+^ population (Figure 6E, and Figures S4B and S4C in Text S1). Neutrophil numbers, quantified by Sudan Black staining, peaked at 72 hpi (2001±312 cells/larva compared to 945±234 cells/larva in uninfected controls) (Figure 6F); both mCherry^+^ and mCherry^−^ neutrophils increased (Figure 6D and *not shown*). Nevertheless, neutrophil distribution was not obviously perturbed: they did not accumulate at infection foci and were absent from the CNS, like in uninfected fish [24]. Interestingly, knockdown of IFN receptors blocked neutrophil increase, indicating that it is dependent on the IFN response (Figure 6G).

### Zebrafish neutrophils play a key role in the control of CHIKV infection

We next addressed the role of neutrophils, macrophages and hepatocytes in the control of CHIKV infection by cell depletion strategies.

**Figure 7:**
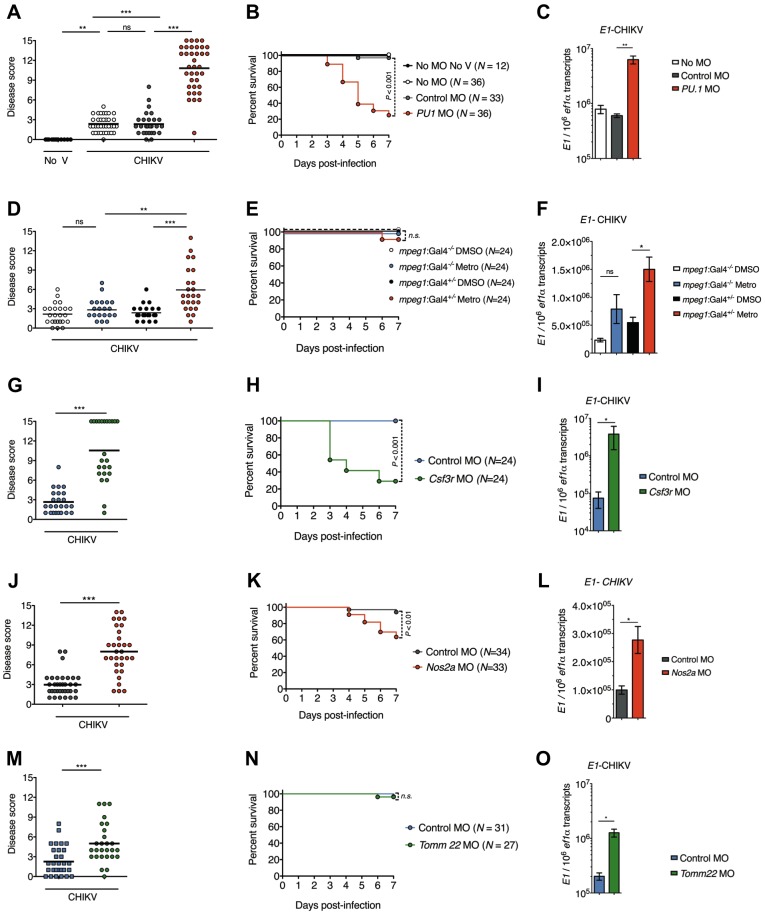
Neutrophils are key ifnφ1-producing cells. (A–C) Effect of myeloid cell depletion (*PU.1* MO, 3 independent experiments), (D–F) of macrophage depletion (metronidazole treatment of *mpeg1:Gal4/UAS:NfsB-mCherry* fish, 2 independent experiments), (G–I) of neutrophil-biased depletion (*csf3r* MO, 2 independent experiments), (J–L) of impairment of emergency granulopoiesis (*nos2a* MO, 2 independent experiments) and (M–O) of hepatocyte depletion (*Tomm22* MO, 2 independent experiments) on CHIKV infection. (A, D, G, J and M) Disease score at 3 dpi. (B, E, H, K and N) Survival of infected zebrafish. Data pooled from the independent experiments. (C, F, I, L and O) qRT-PCR of viral *E1* transcripts at 24 hpi. Mean ± s.e.m of 6 pools of 5 larvae from 2 (C, F, L) or 1 (I, O) independent experiments. (***P < 0.001; **P < 0.01; *P < 0.05; ns - not significant).

First, we blocked myelopoiesis by knocking down PU.1/spi1 (AF321099), resulting in reduced neutrophil and, even more deeply, macrophage populations [37] (Figures S5A and S5B in Text S1; note that head images reflect the impact on mature cells, while tail images include the hematopoietic region to assess depletion of precursors). PU.1 knockdown dramatically increased disease severity (disease score of 10.8±3.4 compared to 2.3±1.6 in control morphants) and mortality (Figures 7A and 7B), and correlated with an increase in viral transcripts (Figure 7C). Therefore, myeloid cells largely control CHIKV in zebrafish.

To distinguish the roles of these two leukocyte types, we first selectively depleted macrophages with a transgenic drug-inducible cell ablation system [38] (Figures S5C and S5D in Text S1). Macrophage-depleted CHIKV-infected larvae exhibited a small increase in disease severity (disease score of 4.8±2.6 compared to 2.3±1.5 in control transgenics) (Figure 7D) but almost no mortality (Figure 7E), despite modestly increased virus amounts (Figure 7F). This suggests that macrophage depletion plays a minor role in the phenotype of PU.1 morphants.

Comparable specific depletion of neutrophils was not available, however *csf3r*/*gcsfr* (NM_001113377) knockdown has been shown to affect neutrophil populations more than macrophages [39]. Indeed, at 3 dpf, our *csf3r* morphants displayed no significant reduction of *mpeg1* ^+^ macrophage numbers, while *mpx* ^+^ neutrophils were severely depleted (Figures S6A and S6B in Text S1); neutrophil depletion lasted until 6 dpf (Figure S6C in Text S1). In infected animals too, *csf3r* knockdown led to a stronger reduction of neutrophils than macrophages, in contrast to PU.1 (Figure S6D in Text S1). *Csf3r* morphants were highly susceptible to CHIKV, with a high disease score (Figure 7G), mortality starting 3 days after infection (Figure 7H), and strongly increased virus transcripts (Figure 7I).

In addition, we attempted to block the increase in neutrophil numbers by knocking down *nos2a* (zebrafish iNOS) (NM_001104937), a strategy recently described to block infection-induced granulopoiesis in a bacterial infection system [40]. The neutrophil population was not reduced in *nos2a* morphants before the infection (*not shown*), but its increase was effectively prevented (Figure S6E in Text S1), and this was associated with increased disease scores (Figure 7J), mortality starting at 4 dpi (Figure 7K), and an increase in viral transcripts (Figure 7L).

Altogether, these experiments provide independent and convergent evidence consistent with neutrophils being the major population controlling CHIKV, in agreement with their predominance among *ifnφ1*-expressing leukocytes (Figure 6C).

Finally, transient hepatocyte depletion using a *Tomm22* (NM_001001724) MO [41] (Figure S7 in Text S1) also led to higher disease severity and more virus production (Figures 7M and 7O) but no increased mortality (Figure 7N), indicating that hepatocytes do not play a role as important as leukocytes in controlling CHIKV.

## Discussion

In this study, we establish zebrafish as a new model for the study of the pathogenesis of CHIKV. The overall course of viral spread in zebrafish larvae was close to that observed in mammals, with an early peak of viremia followed by a decline, similar targeted cell types, and a critical dependence on the host IFN response for the control of the virus. In addition, the powerful *in vivo* imaging techniques available in zebrafish revealed new features of the infection.

We could image the onset of infection in individual cells throughout the body. Almost all new infected cells appeared during one major wave during the first 24 hours following injection of the virus, with relatively little difference between the various targeted organs. Because we could not detect cells with strong GFP expression before the rise of this first wave of infected cells, we presume it reflects the initial set of cells infected by the inoculated virions. The significant inter-individual variation that we observe may be a consequence of a larger number of susceptible cells than of inoculated virions, resulting in a stochastic initial pattern of infection. The decline of appearance of newly infected cells shortly followed the onset of the host IFN response, suggesting that by the time the initial wave of infected cells produce new infectious virions, the host response has made most other cells refractory to the virus. We also observed and quantified infected cell death events, which typically presented apoptosis characteristics. The timing of death of CHIKV^+^ cells was strongly organ-dependent. The differential survival of infected cells accounted for the apparent shift of tropism towards the brain parenchyma, where infection persisted even after clearance from the rest of the body.

The longer persistence of CHIKV in brains of zebrafish suggests that neurons may constitute a previously overlooked reservoir for the virus. However, this is likely to be mostly the case in infant humans, since encephalitis is a feature of chikungunya disease in newborns rather than in adults. CHIKV potential reservoirs are a matter of conjecture because many patients display chronic arthralgia in the months following CHIKV infection despite resolution of viremia, and it is unclear whether this is due to long-lasting auto-inflammation triggered by the initial infection or to stimulation by persistent virus [8], [9], [10]. In adult macaques, CHIKV was suggested to persist in macrophages, not CNS [19]. In infected neonate mice, CHIKV was not found to persist in the brain [18], [42]. Moreover, in this model, CHIKV was found to infect leptomeningeal and choroid plexus cells, but not brain parenchyme. Yet, mouse brain parenchymal cells may be infected by CHIKV, as shown after intranasal infection [43] or on primary cell cultures [17].

The zebrafish model also allowed us to dynamically image and FACS-sort the cells that are responding to the virus by expressing the *ifnφ1* gene. Based on gene expression profile, morphology, and co-expression of the *mpx:GFP* transgene, two main populations were shown to express the *ifnφ1:mCherry* transgene: neutrophils and hepatocytes. Interestingly, while both *irf7* and *irf3* are ISGs in fish [44], and therefore expected to be induced in all cells of infected fish, *irf7* was expressed at a higher level in sorted mCherry^+^ than mCherry^−^ cells. This would be consistent with constitutively higher expression of *irf7* by cells specialized in *ifnφ1* expression in zebrafish – mirroring key properties of plasmacytoid dendritic cells of mammals [45]. The cell types, however, were different. Although not viewed as a specialized source of IFN, hepatocytes have been found to be prominent producers in some cases, for example during Thogoto virus infection of a mouse IFNβ reporter cell line *in vitro* [46]. By contrast, neutrophils are so far not considered to represent an important source of IFN [47], [48]. Nevertheless, in zebrafish larvae, neutrophils were found to represent 80% of *ifnφ1*-expressing leukocytes. In this respect, it should be stressed that our main marker, *mpx*, not entirely neutrophil-specific in mammals, has been shown to be strictly neutrophil-specific in zebrafish [23], [24]. In addition, our depletion experiments were consistent with neutrophils being a key population controlling CHIKV infection in zebrafish, whereas macrophages and hepatocytes made a minor contribution to this control. Macrophage depletion having little consequences, no synergy of macrophages and neutrophils seems required to control CHIKV. However, until a truly neutrophil-specific depletion method becomes available in zebrafish, we cannot rule out the possibility of a significant additive contribution of a minor *csf3r*-dependent macrophage subpopulation to that of neutrophils; compensation mechanisms following depletion are also an important caveat to consider.

Besides IFN production, other mechanisms may be responsible for the observed protective role of myeloid cells, especially neutrophils, against CHIKV pathogenesis. The role of neutrophils in protecting against viral infections is not fully deciphered [49]. Neutrophil extracellular traps (NETs) were recently shown to protect host cells from myxoma virus infection in mice [50] and to capture HIV-1 and promote its elimination through the action of myeloperoxidase and α-defensin in humans [51]. Zebrafish neutrophils, which share many functional characteristics with their human counterparts, including the production of NETs [52], avidly engulf bacteria on surfaces [53] and scavenge dying infected cells in mycobacterial disease [54], but their function during viral infection was so far unknown. It will be worth further studying the role of zebrafish (and human) neutrophils in sensing of CHIKV-infected cells and the mechanisms mediating viral clearance.

Neutrophil numbers were increased with CHIKV infection, a response we found to be dependent on the IFN response. This was contrary to our expectations, as acute IFN induction by viral infection is known to cause granulocytopenia [55], and even in fish, granulocyte numbers were found to be reduced during a viral infection [56]. Interestingly, neutrophilia has been reported in CHIKV-infected humans with a high viral load [57], suggesting that CHIKV may stimulate neutrophils in an unusual manner. Remarkably, this increase was found to depend on *nos2a* (zebrafish iNOS), as had been observed in a *Salmonella* infection model in zebrafish [40]. Depending on the experiment settings, iNOS has been found to favor [58], [59] or counteract [60] neutrophil infiltration in inflamed organs in mice. It would be worth investigating the contribution of iNOS to the inflammatory response induced by CHIKV in mammals.

Comparing patterns of infection and of IFN response, it may be significant that virus persistence - dictated by survival of infected cells - was inversely correlated with local production of IFN. The organ where infected cells died fastest was the liver, which was also a major local source of IFN. Conversely, infected cells persisted much longer in the brain, an organ from which neutrophils are excluded, whereas they patrol other tissues in zebrafish [24]. Assessing the relative contribution of the cell autonomous – such as autophagy [61] – and non-cell autonomous (mostly, IFN-driven) events underlying sensitivity of the cells to the cytopathic effect of CHIKV *in vivo* will be one of our future goals. IFNs have been shown to induce apoptosis of virus-infected cells [62]. It is possible that infected brain neurons and glial cells persist due to the blood brain barrier (BBB) blocking IFN access to this organ. Zebrafish brain endothelial cells express BBB markers Claudin 5 and ZO-1 as early as 3 dpf and brain parenchymal vessels are impermeable to horseradish peroxidase (44 kDa) and rhodamine-dextran (10 kDa) at this stage [63]. It is therefore likely that zebrafish IFNφs (∼20 kDa) cannot reach the brain parenchyma, which would prevent brain-infected cells from undergoing apoptosis. It has also been suggested that less “renewable” tissues and cells, such as post-mitotic neurons, respond to type I IFNs differently from other cell types [62].

Imaging studies detailing the dynamics of single virus-infected cells *in vivo* are very recent and remain scarce [64], [65], [66]. The zebrafish model offers the unique opportunity to visualize and characterize in real time the rise and death of infected cells, throughout the body. To our knowledge, this study represents the first analysis of the fate of single virus-infected cells in a whole organism. Combined with the ability to image IFN-producing cells and to perform host gene silencing, mutagenesis or drug screening, our work establishes the zebrafish as a new valuable host for the study of human pathogenic viruses.

## Materials and Methods

### Ethics statement

All animal experiments described in the present study were conducted at the Institut Pasteur according to European Union guidelines for handling of laboratory animals (http://ec.europa.eu/environment/chemicals/lab_animals/home_en.htm) and were approved by the *Direction Sanitaire et Vétérinaire de Paris* under permit #B-75-1061.

### Fish lines and husbandry

Zebrafish embryos were raised as previously described [67], [68]. Wild-type AB fish were initially obtained from ZIRC (Eugene, OR, USA). The following transgenic lines were used: *Tg(gata1a:DsRed)^sd2^* [69], *Tg(elavl3:EGFP)^knu3^* [70] referred to as *HuC:GFP* in the text, *Tg(gfap:EGFP)^mi2001^* [71], *Tg(fabp10:dsRed)^gz4^* [72], *Tg(mpx:EGFP)^i114^* [73], *Tg(mpeg1:mCherry)^gl23^* and *Tg(mpeg1:Gal4FF)^gl25^* [74], and *Tg(UAS-E1b:Eco.NfsB-mCherry)^c26^* [38] referred to as *UAS:NfsB-mCherry* in the text. For imaging purposes, embryos were generally raised in 0.003% 1-phenyl-2-thiourea (PTU) from 24 hpf onwards to prevent melanin pigment formation.

### Virus

CHIKV was produced on BHK cells. CHIKV-115 is a clinical strain isolated in 2005 from a young adult in La Réunion [2] and its entire genome sequence is available (#AM258990). This virus has been passaged three times since cloning. CHIKV-GFP corresponds to the CHIKV-LR 5′GFP virus generated by insertion of a GFP-encoding sequence controlled by the CHIKV subgenomic promoter between the two main genes of the CHIKV genome, using the LR backbone (#EU224268) derived from the OPY1 strain, a 2006 clinical isolate from La Réunion; GFP expression has been found to be retained in >80% infected cells for up to 8 serial passages in mammalian or mosquito cells [3]. The CHIKV-GFP virus we used previously went through two to three passages.

### Generation of Ifnφ1 reporter transgenics

We generated two independent lines of ifnφ1 reporter transgenics, *Tg(ifnphi1:mCherry)^ip1^* and *Tg(ifnphi1:mCherry)^ip2^* with indistinguishable transgene expression (*not shown*), and both are referred here as *ifnφ1:mCherry* fish. The 6.5 kb SpeI-PstI fragment from PAC clone BUSMP706A0151Q01 (IMAGENE) covering the *ifnφ1* promoter was cloned ahead of the ORF for a farnesylated version of mCherry in a Tol2 derivative vector to yield vector pTol2pIFNL1mC-F. The fragment includes exon 1 including the first codons of the zebrafish *ifnφ1* ORF. This construct was co-injected with *tol2* mRNA into 1-cell stage eggs of AB origin [75].

### CHIKV infection and disease score

Injections and handling of larvae were performed as described [68]. Briefly, zebrafish larvae aged 70–72 hpf were inoculated by microfinjection of ∼10^2^ TCID50 CHIKV (∼1 nl of supernatant from infected BHK cells, diluted to 10^8^ TCID50/ml) in the caudal vein or aorta. Larvae were then distributed in individual wells of 24-well culture plates, kept at 28°C and regularly inspected with a stereomicroscope. Clinical signs of infection were assessed first on aware animals, which were then anesthetized for better observation. Quantitative assessment of the clinical status was based on a precise list of criteria (*see* Table S2 in Text S1) assessed blindly, yielding a disease score ranging from 0 (no disease sign) to 15 (dead or terminally ill).

### CHIKV titration

Infected larvae were snap-frozen and kept at −80°C before homogenization in ∼100 µl of medium; samples were then titrated as TCID50/larva on Vero cells [13].

### qRT-PCR

RNA extraction, cDNA synthesis and quantitative PCR were performed as previously described [34]; externally quantified standards were included to provide absolute transcript amounts. The following pairs of primers (sense and antisense) were used: *GFP*: CCATCTTCTTCAAGGACGAC and CGTTGTGGCTGTTGTAGTTG; *ef1α*: GCTGATCGTTGGAGTCAACA and ACAGACTTGACCTCAGTGGT; *ifnφ1* (secreted isoform): TGAGAACTCAAATGTGGACCT and GTCCTCCACCTTTGACTTGT; *ifnφ3*: GAGGATCAGGTTACTGGTGT and GTTCATGATGCATGTGCTGTA; *viperin*: GCTGAAAGAAGCAGGAATGG and AAACACTGGAAGACCTTCCAA; *E1-CHIKV*: AARTGYGCNGTNCAVTCNATG and CCNCCNGTDATYTTYTGNACCCA (these primers match positions 10921–10943 and 11167–11189, respectively, of the CHIKV genome acc#AM258990, and include degenerate bases, labeled according to the IUPAC convention, making them indifferent to silent mutations); *irf3*: GAGCCAAATCTGGCGACATT and GGCCTGACTCATCCATGTT; *irf7*: TCTGCATGCAGTTTCCCAGT and TGGTCCACTGTAGTGTGTGA; *mpx*: ATGGAGGGTGATCTTTGA and AAGCTATGTGGGATGTGA; *mpeg1*: CCCACCAAGTGAAAGAGG and GTGTTTGATTGTTTTCAATGG; *fabp1a*: AGACAGAGCTAAAACTGTGGT and AGCTGAGAGTGTTACTGATAG; *mCherry*: CCCGCCGACATCCCCGACTA and GGGTCACGGTCACCACGCC. To normalize cDNA amounts, we used the housekeeping gene *ef1α* transcripts, except in specified cases where results were normalized to viral burden using *E1-CHIKV*.

### Whole-body *in vivo* imaging

Larvae were anesthetized and laid on the bottom of an agarose-coated, sealed Petri dish, and imaged as described [34]. To assess efficiency of depletion strategies, *Z*-stacks with 22 µm steps of anesthetized larvae were taken with a Leica Z16 APO A macroscope and quantification performed using ImageJ software. For Figures S6A and S6B in Text S1 quantification was performed as described before [76].

### Time-lapse *in vivo* imaging

For *in vivo* time-lapse imaging, 4–6 larvae, anaesthetized with 112 µg /ml tricaine, were laterally positioned and immobilized in ∼1% low melting point agarose in the center of a 54-mm plastic bottom Petri dish, then covered with 2 ml water containing tricaine. Multiple field transmission and fluorescence imaging was performed using a Nikon Biostation IMQ, using a 10× objective (NA 0.5) and a DSQi camera. Imaging was typically performed at 26°C and *Z*-stacks with 10 µm steps were acquired at least every 30 minutes. Imaging sessions typically lasted 6–24 hours; control uninfected larvae were always included. Cell emergence and death data were concatenated from multiple imaging sessions covering the 0 to 72 hpi time frame.

### Whole-mount immunohistochemistry

IHC was performed as described [77]. Primary antibodies used were: mouse mAb to alphavirus capsid (1∶200) [78], rabbit polyclonal to DsRed (1∶300, Clontech) which also labels the mCherry protein, mouse monoclonal to GFP (1∶500, Invitrogen), chicken polyclonal to GFP (1∶500, Abcam), mouse monoclonal (FIS 2F11/2) to gut secretory cell epitopes (1∶400, Abcam). Secondary antibodies used were: Cy3-labeled goat anti-rabbit or anti-mouse IgG (1∶300, Jackson Immunoresearch), Alexa 488-labeled goat anti-mouse or anti-chicken (1∶500, Invitrogen). Nuclei were stained for 30 min at room temperature with Hoechst 33342 at 2 µg/ml (Invitrogen).

### Imaging of fixed embryos

Fixed embryos were progressively transferred into 80% glycerol before imaging. Confocal images of IHC-processed fish were taken with a Leica SPE inverted confocal microscope equipped with a 16× (NA 0.5), 63× (NA 1.30) oil immersion objectives and a 10× (NA 0.30) dry objective. Images of larvae stained by WISH or Sudan Black B were taken with a Leica MZ16 stereomicroscope using illumination from above. Whole-body images of IHC-treated larvae were taken with a Leica Z16 APO A macroscope. Images were processed with the LAS-AF (Leica), ImageJ and Adobe Photoshop softwares. Cells with amoeboid morphology were scored as “leukocytes”.

### Morpholino injections

Morpholino antisense oligonucleotides (Gene Tools) were injected into 1–4-cell stage embryos as previously described [68]. *crfb1* splice morpholino (CGCCAAGATCATACCTGTAAAGTAA) (2 ng) was injected together with *crfb2* splice morpholino (CTATGAATCCTCACCTAGGGTAAAC) (2 ng), knocking down all type I IFN receptors [26]. Other morpholinos: *mavs* splice morpholino (ATTTGAATCCACTTACCCGATCAGA) (4 ng); *tomm22* translation morpholino [41] (GAGAAAGCTCCTGGATCGTAGCCAT) (2 ng); *pu.1* translation morpholino (GATATACTGATACTCCATTGGTGGT) [37] (20 ng in 2 nl); *csf3r* translation morpholino (GAAGCACAAGCGAGACGGATGCCAT) [74] (4 ng); *nos2a* splice morpholino (ACAGTTTAAAAGTACCTTAGCCGCT) [40] (6 ng). Control morpholinos with no target: #1 (GAAAGCATGGCATCTGGATCATCGA) (2–6 ng); #2 (TACCAAAAGCTCTCTTATCGAGGGA) (20 ng); #3 (CCTCTTACCTCAGTTACAATTTATA) (4 ng).

### Embryo dissociation and FACS sorting

Embryo dissociation was performed as described elsewhere [79]. Sorted cells were collected in lysis buffer and RNA was extracted using a RNAqueous Micro kit (Ambion). Cell preparations were performed in a BL3 facility; the cell sorter, located under a plastic tent within a BL2 facility, was flushed for several hours with diluted bleach following the sorting.

### Whole-mount *in situ* hybridization

WISH was performed as described before [80], with a hybridization temperature of 55°C. To generate the *ifnφ1* antisense probe, we RT-PCR amplified a 503 bp fragment of zebrafish *ifnφ1* cDNA from CHIKV-infected larvae using a T3-modified antisense primer (GAATTCATTAACCCTCACTAAAGGGAGATTGACCCTTGCGTTGCTT) and a normal sense (TCTGCAGAGTCAAAGCTCTG). PCR products were purified with QIAquick PCR purification kit (Qiagen) and the probe was transcribed *in vitro* with T3 polymerase (Promega). Unincorporated nucleotides were removed by purification on NucAway spin columns (Ambion).

### Sudan Black B staining

Neutrophil granules were stained as in [24], allowing neutrophils to be counted easily with a dissecting scope.

### Macrophage depletion

Metronidazole-mediated depletion was performed as described in [38]. Briefly *Tg(mpeg1:Gal4FF)^gl25/−^* [74] were crossed to *Tg(UAS-E1b:NfsB-mCherry)^c264/c264^* [38] to generate double-positive transgenics and single-positive sibling controls. Embryos were placed, from 48 hpf to 70 hpf, in a 10 mM Metronidazole, 0,1% DMSO solution to induce specific depletion of NfsB-mCherry-expressing macrophages. Embryos were then rinsed 3× with embryo water.

### Statistical analysis

To evaluate difference between means, a two-tailed unpaired *t*-test or an analysis of variance (ANOVA) followed by Bonferroni's multiple comparison test was used, when appropriate. Normal distributions were analyzed with the Kolmogorov-Smirnov test. Non-Gaussian data were analyzed with a Kruskal-Wallis test followed by Dunn's multiple comparison test. *P*<0.05 was considered statistically significant (symbols: ***P<0.001; **P<0.01; *P<0.05). Survival data were plotted using the Kaplan-Meier estimator and log-rank tests were performed to assess differences between groups. Statistical analyses were performed using Prism software.

## Supporting Information

Movie S1 **Emergence of new CHIKV-GFP infected cells.** Time-lapse imaging of a CHIKV-GFP-infected larva with time post-infection (pi) shown on top left corner. Overlay of transmitted light and wide-field GFP fluorescence; 10× objective; anterior to left, dorsal to top, lateral orientation with some dorsal tilt. The emergence of new infected cells, detected by the onset of GFP fluorescence, is indicated by arrows; only cells in the focal plane are shown. Green arrows point to liver cells, yellow arrows to head mesenchyme or gill cells, and magenta arrows forother cells. The death of a liver cell is shown with a red arrow.
(MOV)

Movie S2 **Death of CHIKV-GFP infected cells.** Time-lapse imaging of CHIKV-GFP infected larvae; time post-infection (pi) shown on top left corner. Overlay of transmitted light and wide-field GFP fluorescence; 10× objective; anterior to left, dorsal to top. Death of GFP^+^ cells shown with red arrows. First sequence (seconds 1–4): head view, death of epidermal cells over the eye and head mesenchymal cells; second sequence (seconds 4–8): liver region, death of hepatocytes; third sequence (seconds 8-3): tail tip region, death of fin fibroblasts; in this sequence a black arrowhead follows a leukocyte that likely engulfs a dying cell.
(MOV)

Movie S3 **Increase of Ifnφ1-expressing leukocytes during the first days of infection.** Time-lapse imaging of a CHIKV-GFP infected *ifnφ1:mCherry^+^* larva; time post-infection (pi) shown on top left corner. Overlay of GFP (in green) and mCherry (in red) spinning-disk confocal fluorescence images; 10× objective; anterior to left, dorsal to top. This region has been chosen for imaging because it is rich in leukocytes, since the main hematopoietic region at this stage lays immediately caudal to the urogenital opening. Note that the growth of the larva causes some movement of the imaged region towards the right and bottom of the field.
(MOV)

Text S1 **File containing Figures S1–S7 and Tables S1–S2, with legends.**
(PDF)
