# Supplementary material for: Real-Time Whole-Body Visualization of Chikungunya Virus Infection and Host Interferon Response in Zebrafish
Source: PLoS Pathog. 2013 Sep 5;9(9):e1003619. doi: 10.1371/journal.ppat.1003619 (PMC3764224; doi:10.1371/journal.ppat.1003619)
Supplement: Text S1 — File containing Figures S1–S7 and Tables S1–S2, with legends. (PDF) [file ppat.1003619.s004.pdf]

Palha et al.,

"Real-time Whole-body Visualization of Chikungunya Virus Infection and Host Interferon Response in Zebrafish"

Text S1, including:

Supplementary Figures S1-S7

Tables S1-S2

Figure S1

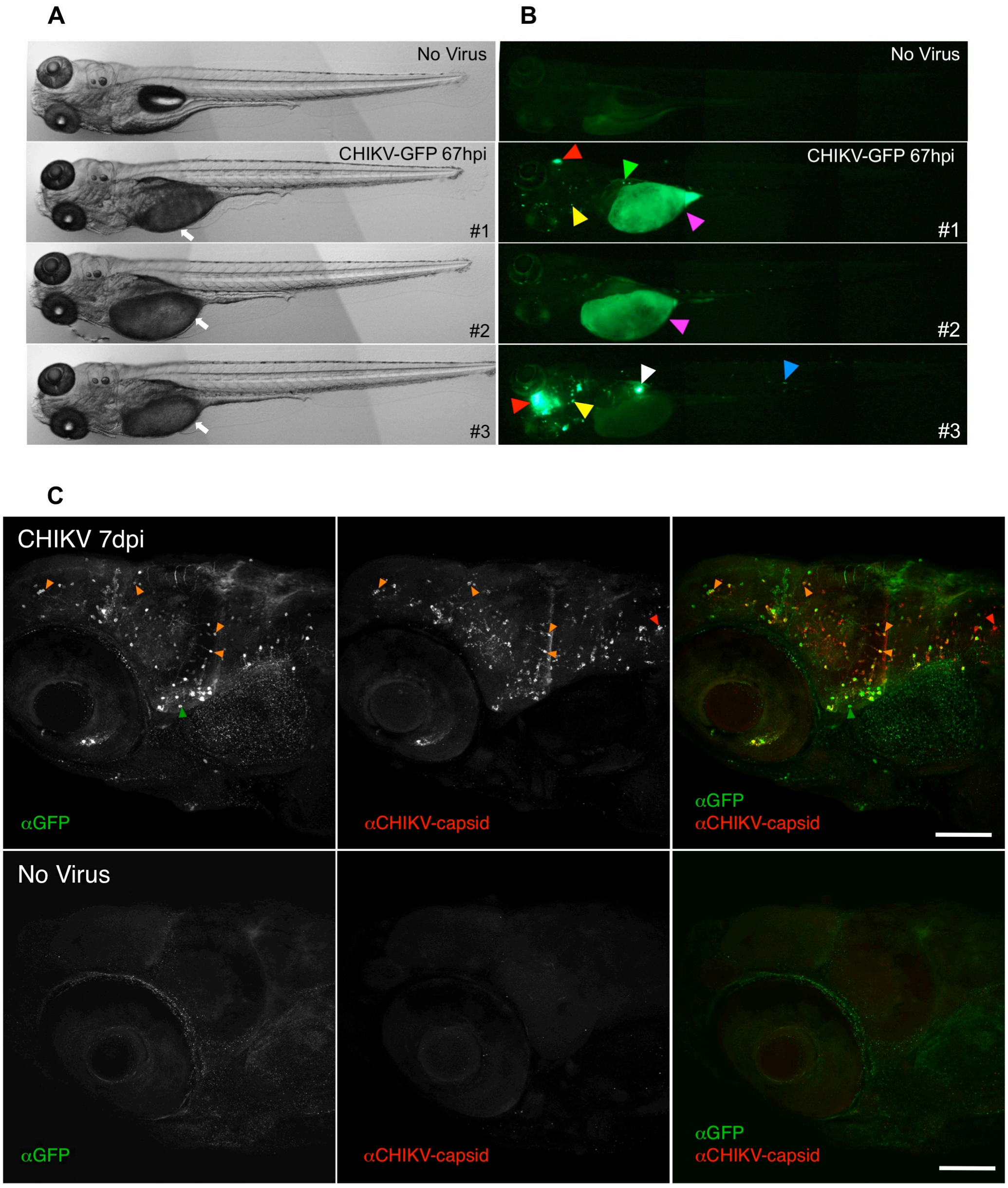

Figure S1. CHIKV tropism variation between individual zebrafish and persistence in brain. (A) Transmission and **(**B**)** Fluorescence live images of 3 CHIKV-GFP-infected zebrafish 67 hours post-infection (hpi) and an uninfected control above (No Virus). CHIKV infection is shown in the brain, liver, head mesenchyme, yolk syncitium, muscle and swim bladder (red, green, yellow, magenta, blue and white arrowheads, respectively). White arrows point opacification of the yolk in infected fish. (C) Confocal images of IHC-processed CHIKV-GFP infected zebrafish at 7 days post-infection and an uninfected control (below). GFP staining on the left grayscale single fluorescence image and in green in merged image (right). Capsid staining in middle image and in red in the merged image (right). Infection is seen in neurons of the CNS. Orange arrows point to double-positive cells, green and red arrow point single GFP^+ and single $capsid^+$ cells, respectively. Note that the relative levels of GFP and capsid signal are strongly dependent on the depth of the cells because of stronger absorption of shorter wavelengths by tissue. As for all images, anterior to left, dorsal to top; maximal projections, scale bar, 100 µm.

Figure S2

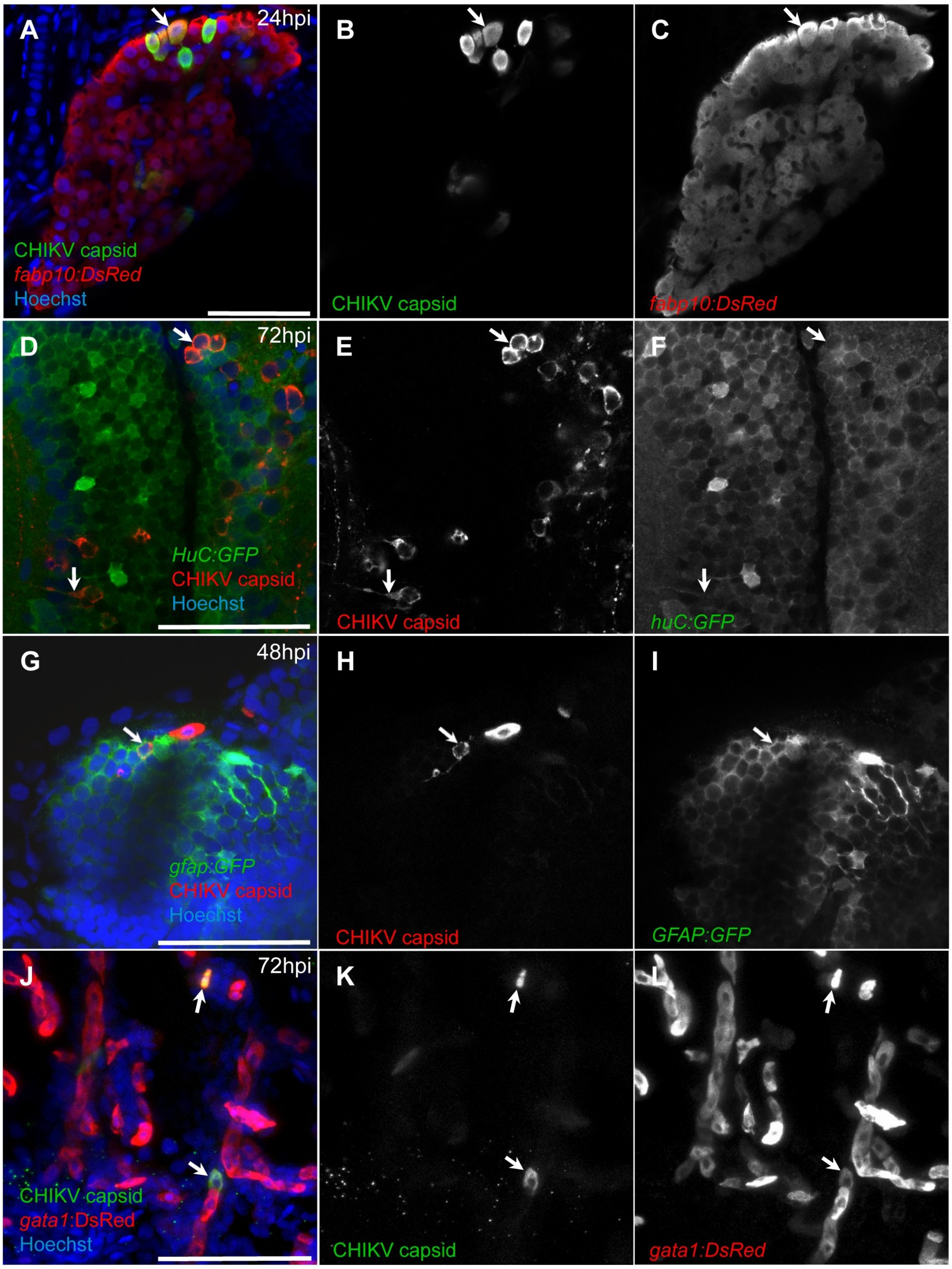

Figure S2. CHIKV infects hepatocytes, neurons, glial cells and erythrocytes in zebrafish. Confocal sections of IHC-processed zebrafish, merged colour images (left column) or grayscale single fluorescence images (middle and right columns). 24 (A-C), 48 (G-I) or 72 (D-F, J-L) hours post infection with CHIKV-GFP (A-C, G-L) or CHIKV-115 (D-I). Viral capsid-specific staining in green (A, L) or red (D, G). *fabp10:DsRed*$^{+}$ hepatocytes and *gata1:DsRed*$^{+}$ erythrocytes were stained for DsRed (red in A, J), *HuC:GFP*$^{+}$ neurons (in cerebellum) and *gfap:GFP*$^{+}$ glial cells (in telecephalon) were stained for GFP (green in D, G). Anterior to left, dorsal to top. Arrows point representative infected cells; scale bars, 50 μm.

Figure S3

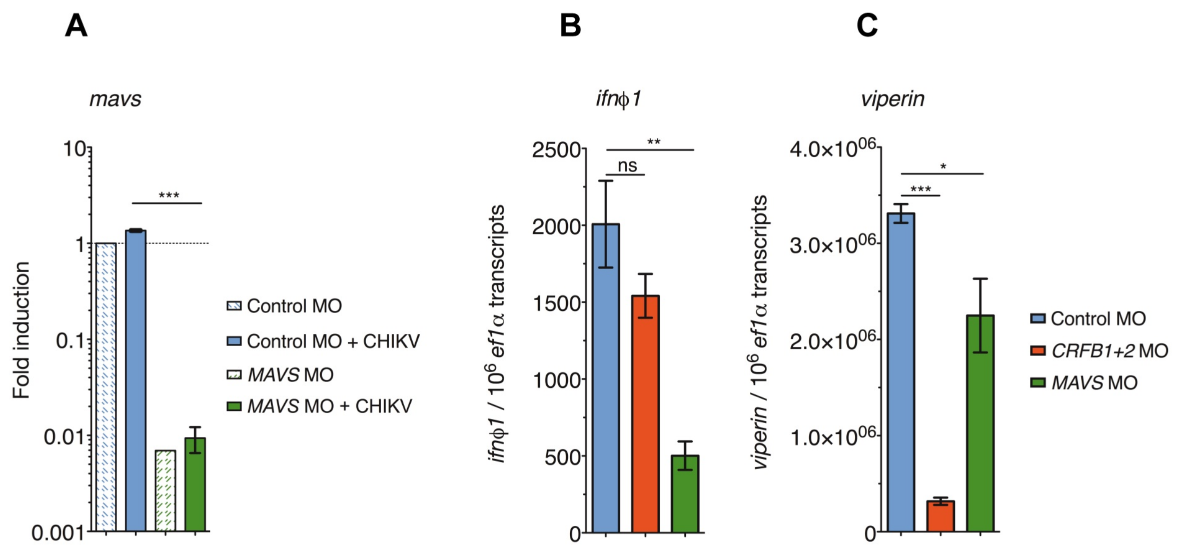

Figure S3. Interferon signaling and virus sensing are inhibited with IFN receptors and MAVS morpholino-mediated knockdown, respectively. qRT-PCR of *MAVS* (A), of *ifnφ1* (B) and of the IFN-stimulated gene *rsad2*/*viperin* (C), 24 hours post CHIKV-GFP infection, in *CRFB1*/*CRFB2*, *MAVS* or control morphant larvae. Absolute quantifications shown in (B, C); relative expression to Control MO of properly spliced *MAVS* transcripts (normalized to *ef1α* levels) in (A). Data represent mean ± s.e.m of 3 pools of 5 larvae, except for uninfected controls in (A), where results are shown for a single pool. (****P*<0.001; ***P*<0.01; **P*<0.05; ns - not significant).

Figure S4

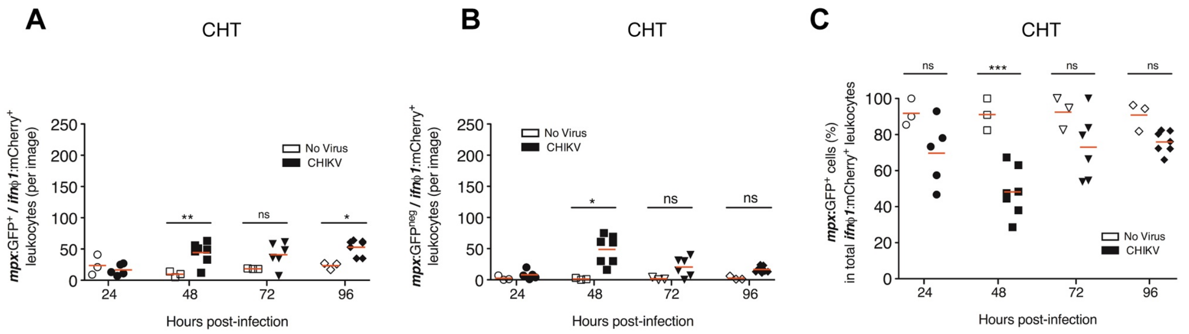

Figure S4. ***Ifnϕ1*-expressing leukocytes in the caudal hematopoietic tissue (CHT).** Double transgenic *mpx:GFP/ifnϕ1:mCherry* fish, infected or not, were IHC-labeled for mCherry (red) and GFP (green), and the CHT imaged with a confocal microscope. (A) Numbers of $mCherry^+$ neutrophils (i.e., GFP^+ cells) per field. (B) Numbers of other $mCherry^+$ leukocytes (GFP^-) per field. (C) Percentage of neutrophils (GFP^+) among $mCherry^+$ leukocytes, per field. *N*=3 (No Virus) or N=5-7 (CHIKV-115); (****P*<0.001; ***P*<0.01; **P*<0.05 ; ns - not significant).

Figure S5

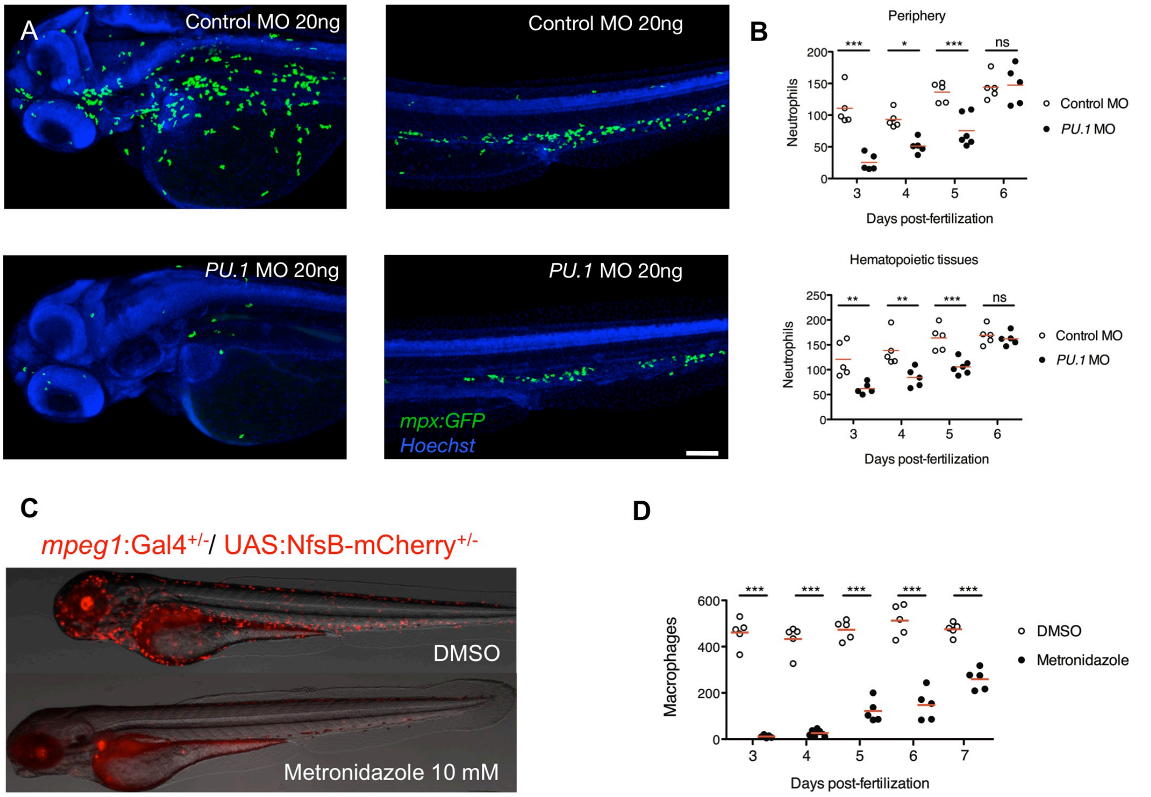

Figure S5. Efficiency of myeloid cells and macrophage depletion strategies. (A) Myeloid cell depletion: neutrophil reporter *mpx:GFP* fish, injected at the 1-cell stage with *PU.1* or control morpholino, were IHC-labeled for GFP (green). Confocal imaging at 72 hpf, maximal projections, scale bar 50 µm, nuclei in blue. Anterior region shown on the left and uro-genital/CHT region on the right. Two representative animals of each group, controls on top and *PU.1* morphants shown below. (B) Number of neutrophils (*mpx*:GFP^{+} cells) in hematopoietic tissues and other tissues (Periphery) upon *PU.1* MO injection, *N*=5 larvae per group. (C, D) Macrophage depletion: double transgenic *mpeg1:Gal4FF/UAS:NfsB-mCherry* fish were treated with 10mM metronidazole from 48 to 70 hours post fertilization (hpf) to specifically deplete Nfsb-expressing cells, ie macrophages (*mpeg1*$^{+}$ cells), also labelled by mCherry fluorescence. (C) Overlay of transmission and red fluorescence stereomicroscope images of representative live *mpeg1:Gal4FF*$^{+/-}$*/UAS:mCherry-NfsB*$^{+/-}$ 72 hpf fish following DMSO only or metronidazole treatment. (D)**,** Number of macrophages (*mpeg1*:mCherry^{+} cells) upon metronidazole-mediated depletion. *N*=5 larvae per group (****P*<0.001; ***P*<0.01; **P*<0.05 ; ns - not significant).

Figure S6

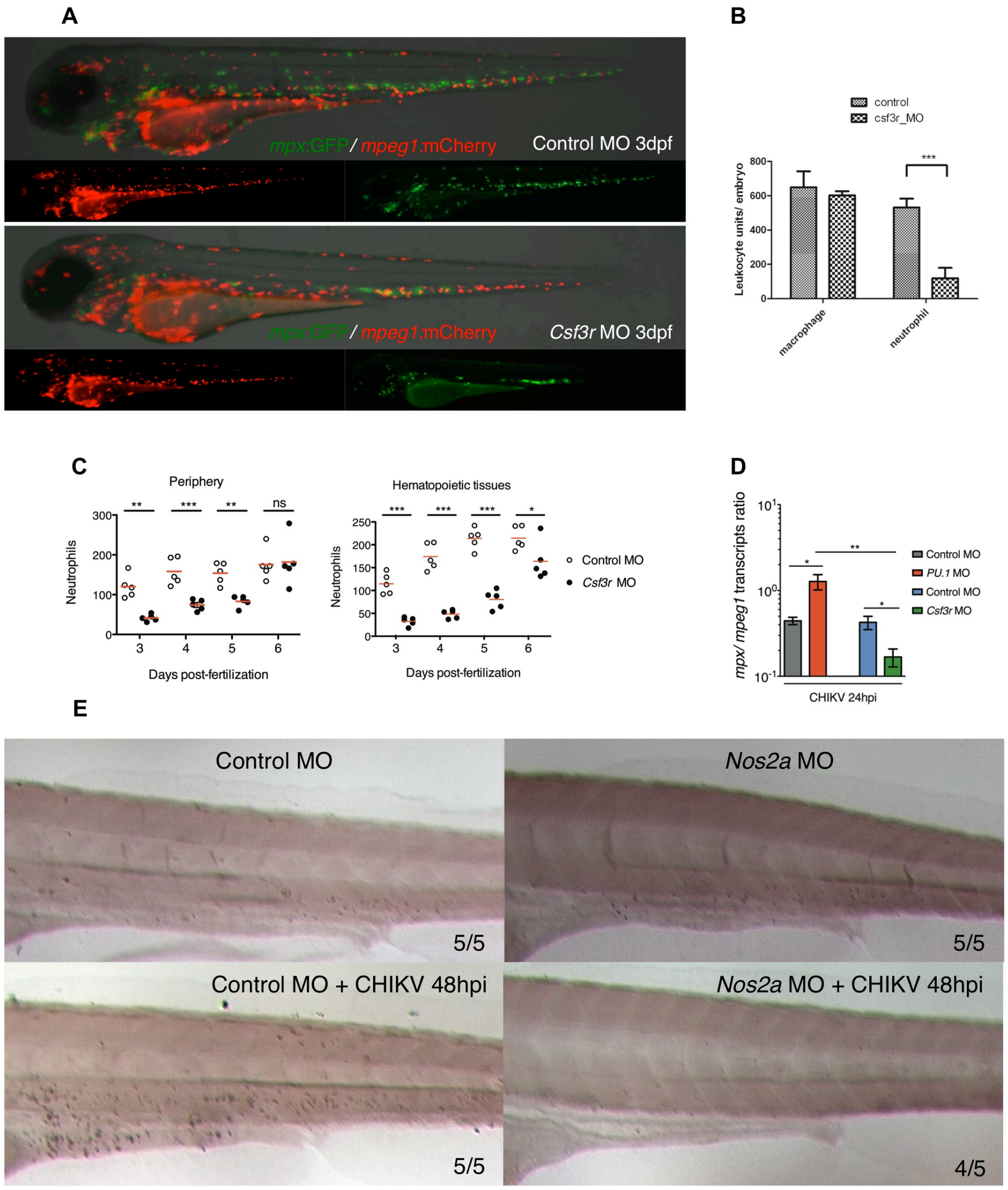

Figure S6. Efficiency of neutrophil cell depletion strategies. (A-D) *Csf3r* MO effect in neutrophil and macrophage populations. (A) Merged fluorescence and transmission images, or fluorescence only (below), of 3dpf double transgenic *mpx*:GFP/*mpeg1*:mCherry larvae injected with control morpholino #3 (top) or *Csf3r* morpholino (bottom). (B) Numbers of neutrophils and macrophages at 3dpf. Quantification displayed in leukocyte units as described elsewhere [76]. (C, D) *Csf3r* MO-mediated neutrophil depletion efficiency in experiments of Figures 7G-I. (C) Neutrophil numbers in hematopoietic tissues and other tissues (Periphery) upon *Csf3r* MO injection, *N*=5 larvae per group. (D) qRT-PCR of *mpx* and *mpeg1* transcripts 24 hours post CHIKV-GFP infection, in *PU.1*, *Csf3r* or control morphant larvae. A ratio of *mpx* to *mpeg1* transcripts (normalized to *ef1α* levels) is shown. Data represent mean ± s.e.m of 3 pools of 4 larvae. (E) Transmission images of Sudan Black B-stained neutrophils in the CHT of CHIKV-GFP infected (bottom) or uninfected (top) larvae at 48hpi. Control morphants to the left and *nos2a* morphants to the right. As for all images, anterior to left, dorsal to top. Numbers represent frequency of larvae with displayed phenotype out of 5 fish. (***$P<0.001$; **$P<0.01$; *$P<0.05$; ns - not significant).

Figure S7

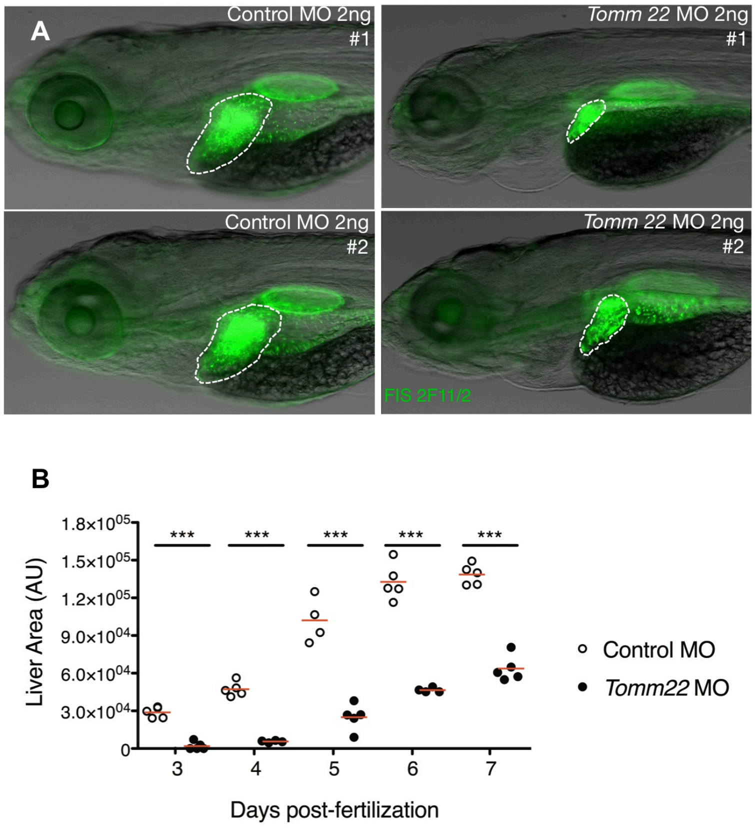

Figure S7. Efficiency of liver cell depletion. (A) Hepatocyte depletion: liver atrophy due to hepatocyte depletion in *tomm22* morphants at 5 dpf. Fish were IHC-labeled with the FIS 2F11/2 antibody that labels biliary duct cells in liver, and secretory enterocytes in gut. Overlay of transmission and fluorescence images of representative fish are shown. Liver delineated with white-dotted line. (B) Liver area in arbitrary units (AU) in *tomm22* morphants. N=5 larvae per group (***P<0.001).

Table S1

Clinical signs of disease at 3 dpi

Clinical sign	No Virus (*N*=24)	CHIKV (*N*=24)	Control MO + CHIKV (*N*=24)	*CRFB1+2* MO + CHIKV (*N*=24)
**Dead**	0	0	0	9
Impaired equilibrium	0	4	2	15
Impaired response to physical stimuli	0	5	0	15
Bent body	0	1	2	8
Abnormal blood flow	0	6	1	15
Abnormal heart beat	0	5	7	15
Edema present	0	7	4	15
No inflated swim bladder	0	3	7	15
Yolk opacification	0	21	24	15

Table S1. Clinical signs of disease at 3 days post-infection (dpi). Frequency of zebrafish displaying a given clinical sign 3 days after CHIKV-GFP infection, in standard animals (no morpholino injected), or IFN-receptor knockdown (*CRFB1+2 MO*) or control morphants. Data pooled from 3 independent experiments.

Table S2

**1) Observation before anesthesia**			
**Is the animal alive?** (*i.e.*, turgid)	**No**: score = 15; **Yes**: consider each of the following criteria, and work out the total score by adding disease points		
Disease points	***0***	***1***	***2***
**equilibrium**	proper upright position	lying on side	
**response to light touch**	quick escape	sluggish escape	no escape
**2) Observation on anesthetized animal**			
**body shape**	straight	slight curve	strong curve
**blood flow**	normal	abnormal (1)	none
**heart beat**	normal	abnormal (2)	none
**edema**	none	1 place & minor	2 places or major
**swim bladder**	medium or large	small	absent
**yolk**	normal (3)	local opaque spots	mostly opaque and/or with protrusions

(1): too slow and/or not in all body parts and/or very few moving cells

(2): low amplitude and/or irregular and/or very slow and/or very quick

(3) clear yolk, or, past 7dpf, fully resorbed

Table S2. Chikungunya disease scorecard. Method used to calculate a disease score to evaluate disease severity after CHIKV infection of zebrafish larvae using a dissecting scope with transmitted light. For each parameter (with the exception of "equilibrium"), a score of 0, 1 or 2 was attributed to a given fish according to the severity of the observed clinical sign. After evaluation of the above listed 8 parameters, all points are added; a maximum total of 15 can be attained (corresponding to death or terminal stage of disease); 0 corresponds to no detectable disease. Scoring was performed in a blind fashion.
